# Association between red cell distribution width and the risk of heart events in patients with coronary artery disease

**DOI:** 10.3892/etm.2015.2244

**Published:** 2015-01-30

**Authors:** WEIMIN LI, XIAOTING LI, MAOFENG WANG, XUAN GE, FEIXIANG LI, BIAN HUANG, JIREN PENG, GUOHONG LI, LIANG LU, ZHUOYUAN YU, JIAOJIAO MA, LIAOHANG XU, MEIJUAN JIN, HONGPING SI, RUGEN WAN

**Affiliations:** 1 Department of Cardiology, Affiliated Dongyang Hospital of Wenzhou Medical University, Dongyang, Zhejiang 322100, P.R. China; 2 Department of Cardiology, Sir Run Run Shaw Hospital, School of Medicine, Zhejiang University, Hangzhou, Zhejiang 310000, P.R. China; 3 Department of Clinical Laboratory Medicine, Affiliated Dongyang Hospital of Wenzhou Medical University, Dongyang, Zhejiang 322100, P.R. China

**Keywords:** red cell distribution width, coronary artery disease, Framingham risk score, heart events

## Abstract

Red cell distribution width (RDW) has been found to be a novel prognostic biomarker in patients with coronary artery disease (CAD); however, the association between RDW and the risk of heart events in patients with CAD is yet to be fully elucidated. Thus, the aim of the present study was to determine whether an elevated RDW was associated with the Framingham risk score (FRS) in patients with CAD. Data were retrospectively collected from Affiliated Dongyang Hospital of Wenzhou Medical University (Dongyang, China). The patients had undergone a coronary angiography and their clinical data were integrated. The patients (male, 260; female, 132) were divided into two groups based on the results of the coronary angiography, namely the CAD (n=283) and control groups (n=109). The FRS was calculated for all the subjects, and complete blood count testing with biochemical measurements was performed. The mean RDW level was 13.7±1.8% in the CAD group and 13.1±1.0% in the control group, while the mean FRS was 9.0±4.9 in the CAD group and 6.4±3.9 in the control group. The RDW and FRS were significantly higher in the CAD group compared with the control group (P<0.001). No statistically significant differences were observed between the groups with regard to the hematocrit, mean corpuscular volume, platelets, glucose, urea, albumin, aspartate aminotransferase, total cholesterol, triglycerides (TG), high-density lipoprotein cholesterol, low-density lipoprotein cholesterol and N-terminal pro-brain natriuretic peptide (P>0.05). The RDW was shown to significantly correlate with the red blood cell (RBC) count (*r*=−0.133, P=0.029), hemoglobin level (*r*=−0.207, P=0.001) and TG level (*r*=0.226, P<0.001) within the laboratory parameters, as well as the FRS (*r*=0.206, P<0.001). In the stepwise multivariate linear regression, which included the RBC count, hemoglobin level, TG level and RDW, the FRS was predicted by hemoglobin (*r*^2^=0.034, P=0.001), TG (*r*^2^=0.059, P<0.001) and RDW (*r*^2^=0.030, P=0.003) parameters. Therefore, a novel association was revealed between higher levels of RDW and an elevated FRS in patients with CAD, which raises the possibility that a simple marker, RDW, may be associated with an increased risk of heart events in CAD patients.

## Introduction

In current clinical practice, one of the most frequently ordered laboratory tests is the complete blood count (CBC). The standard CBC test comprises white blood cell (WBC), red blood cell (RBC) and platelet counts, and their morphological indices. Numerous studies have investigated the efficacy of these hematological CBC parameters in the prediction of disease severity (1) and mortality risk (2). For example, the red cell distribution width (RDW) to platelet ratio has been proposed as a biomarker for liver fibrosis and cirrhosis (3). Furthermore, an increase in RDW has been shown to be associated with mortality and other severe adverse outcomes in cardiac (4–8), renal (9) and infectious diseases (10–12), as well as for individuals in the general population (13,14). Coronary artery disease (CAD) is one of the leading causes of mortality and morbidity (15,16). The gold standard for the diagnosis and selection of therapeutic methods for CAD is an invasive conventional coronary angiography, which outlines the severity and complexity of the CAD (17,18). RDW is a newly identified novel risk marker that has been reported to be a predictor for morbidity and mortality in a variety of cardiovascular conditions (19,20). Previous studies have reported that an elevated RDW is significantly associated with a poor prognosis in patients with heart failure (21) and stable CAD (15). Despite these correlations, a limited number of studies have evaluated the association between RDW and the risk of heart events in patients with CAD. Since measuring these indices and evaluating the associations with disease progression and prognosis is relatively simple and cost-effective, the present study aimed to determine whether an elevated RDW was associated with the Framingham risk score (FRS) in patients with CAD. FRS is a well-established risk assessment tool, with a high degree of concordance between expected and actual event frequencies (22)

## Subjects and methods

### Ethical approval

The study was approved by the Human Investigation Ethics Committee of the Affiliated Dongyang Hospital of Wenzhou Medical University (Dongyang, China). All the patients provided oral informed consent prior to enrolment in the study.

### Subjects and sample collection

Data were retrospectively collected between January 2012 and September 2013 from Affiliated Dongyang Hospital of Wenzhou Medical University. All the patients had undergone a coronary angiography and their clinical data were integrated. The patients (male, 260; female, 132) were divided into two groups based on the results of coronary angiography, namely the CAD (n=283) and control groups (n=109). CAD was defined as stenosis of ≥50% in the left main coronary artery, the left anterior descending artery, the left circumflex coronary artery, the right coronary artery or the main branch of the coronary artery.

All the individuals enrolled in the study, regardless of a CAD diagnosis, exhibited normal hepatic and renal function. Individuals were excluded from the study if they presented with evidence of myocardial infarction, valvular heart disease, left ventricular dysfunction, congestive heart failure, had a history of dysphagia, swallowing and intestinal motility disorders, had untreated thyroid disease, sinus node dysfunction or conduction disturbance, were undergoing estrogen replacement therapy, had an autoimmune disease or had suffered from a recent infection (within the previous three months). Subjects who had a previous history of anemia, were receiving treatment for anemia, including supplementary iron, folate or an erythropoiesis-stimulating agent, or had received a RBC transfusion were also excluded from the study. Furthermore, patients with a known hematological disease, including hemolytic anemia and neoplastic metastases to the bone marrow, or those on iron replacement therapy that may have increased the plasma RDW, were not included in the study.

Baseline clinical and demographic characteristics were obtained from all the patients. A detailed physical examination was performed, which included past medical history, and hematological and clinical data were obtained from the patients. The FRS was calculated by assigning gender-specific points, as recommended by the National Cholesterol Education Program Expert Panel on the Detection, Evaluation, and Treatment of High Blood Cholesterol in Adults (Adult Treatment Panel III) guidelines (23). Hypertension was identified based on previous prescriptions for antihypertensive drugs or when the blood pressure exceeded 140/90 mmHg in at least three measurements. Diabetics were defined as having received a prior prescription of antidiabetic medications or having fasting glucose levels of >7 mmol/l. Current smokers and drinkers were defined as subjects with a positive history of cigarette smoking and alcohol drinking, respectively.

### Laboratory analysis

CBC testing was performed using an automated hematology analyzer XE-2100 (Sysmex Corporation, Kobe, Japan) for clinical purposes during the baseline hospitalization. CBC metrics included the hematocrit, hemoglobin levels, RDW, mean corpuscular volume (MCV), and RBC, platelet (PLT) and total WBC counts. The other biochemical measurements were performed using a Hitachi 7600–120 chemical analyzer (Hitachi, Ltd., Tokyo, Japan). N-terminal pro-brain natriuretic peptide (NT-proBNP) analysis was performed with an automated electrochemiluminescence analyzer (Modular Analytics Cobas e601; Roche Diagnostics GmbH, Mannheim, Germany).

### Statistical analysis

Data are expressed as the mean ± standard deviation or the median with the interquartile range, depending on the distribution. Univariate comparisons of continuous variables were performed using a two-sample independent t-test for normally distributed data or non-parametric Mann-Whitney U-test for non-normally distributed variables. For multiple comparisons of several groups, analysis of variance or a Kruskall-Wallis test was performed. The χ^2^ test was performed for categorical data comparisons. Associations between continuous variables were determined using Pearson’s correlation analysis. Since the levels of aspartate aminotransferase (AST), creatine kinase (CK), creatine kinase-MB (CK-MB), lactate dehydrogenase (LDH), C-reactive protein (CRP) and NT-proBNP were not normally distributed, a logarithmic transformation was used. To determine the variables independently associated with the FRS, a stepwise multivariate linear regression was performed, which included the variables that correlated significantly with the FRS. P=0.05 was set as the threshold for inclusion, while P=0.10 was set as the threshold for exclusion of variables. A two-sided P-value of <0.05 was considered to indicate a statistically significant difference. Statistical analysis was performed using SPSS software 13.0 (SPSS, Inc., Chicago, IL, USA) for Windows.

## Results

### Baseline characteristics

Baseline characteristics of the patients with CAD and the control subjects are shown in Table I. In total, 283 patients were included in the CAD group and 109 individuals were included in the control group, comprising a total of 392 subjects. The mean age was 67±10 years in the CAD group and 62±10 years in the control group; thus, the age was significantly higher in the CAD group (P<0.001). In the CAD group, 20.1% (57/283) of patients were diabetic and 68.9% (195/283) were hypertensive. By contrast, in the control group, 11.0% (12/109) of the patients were diabetic and 39.4% (43/109) were hypertensive. The diabetic and hypertensive rates in the CAD group were significantly higher compared with those in the control group (P<0.05). No statistically significant differences were observed between the CAD and control groups with regard to gender, body mass index (BMI), smoking and drinking (P>0.05).

### Laboratory observations

No statistically significant differences were observed between the groups with regard to the hematocrit, MCV, PLT count, and levels of glucose, urea, albumin, AST, total cholesterol (TC), triglyceride (TG), high-density lipoprotein cholesterol (HDL-C), low-density lipoprotein cholesterol (LDL-C) and NT-proBNP. The mean RDW was 13.7±1.8% in the CAD group and 13.1±1.0% in the control group; thus, the RDW was significantly higher in the CAD group when compared with the control group (P<0.001). The mean FRS was 9.0±4.9 in the CAD group and 6.4±3.9 in the control group; the FRS was significantly higher in the CAD group compared with the control group (P<0.001). Furthermore, patients in the CAD group had significantly higher levels of WBCs, neutrophils, creatinine, CK, CK-MB, LDH and CRP, and lower levels of lymphocytes, RBC and hemoglobin when compared with the levels in the control group. Table II shows the hematological and clinical data of the patients with CAD and the control subjects.

### Hematological and clinical data of the diabetic and hypertensive patients

No statistically significant differences were observed between the two groups with regard to the levels of WBCs, neutrophils, lymphocytes, RBCs, hemoglobin, RDW, creatinine, CK, LDH and CRP. The mean FRS was 8.9±4.2 in the diabetic and hypertensive group, 8.2±4.1 in the diabetic or hypertensive group and 7.9±3.1 in the group with no subjects with diabetes or hypertension. The FRS was significantly different among the three groups (P=0.001). Table III shows the hematological and clinical data for all the subjects, differentiated by diabetic and hypertensive conditions.

### Associations between the RDW and laboratory parameters

**Figure 1:**
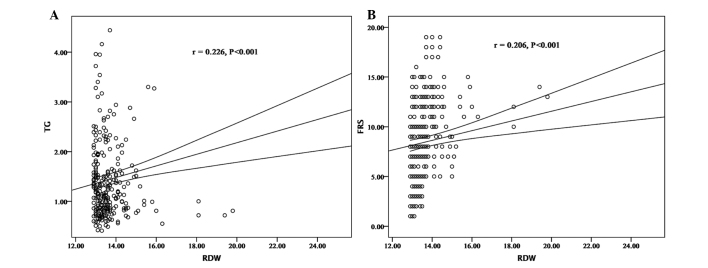
Correlation between RDW and (A) TG and (B) FRS. RDW, red cell distribution width; TG, triglycerides, FRS, Framingham risk score.

Correlations between the RDW and laboratory parameters or the FRS were investigated (Table IV and Fig. 1). The RDW was shown to significantly correlate with the RBC count (*r*=−0.133, P=0.029), hemoglobin level (*r*=−0.207, P=0.001) and TG level (*r*=0.226, P<0.001) within the laboratory parameters, as well as the FRS (*r*=0.206, P<0.001). However, other parameters, including age, BMI, WBC, MCV, PLT, creatinine, TC, HDL-C, LDL-C, CRP and NT-proBNP did not exhibit a statistically significant correlation with the RDW. In the stepwise multivariate linear regression, which included the RBC count, hemoglobin level, TG level and RDW, the FRS was predicted by hemoglobin (*r*^2^=0.034, P=0.001), TG (*r*^2^=0.059, P<0.001) and RDW (*r*^2^=0.030, P=0.003). Table V shows the results from the stepwise multivariate linear regression for FRS in patients with CAD.

## Discussion

In the present study, the RDW and FRS were demonstrated to be significantly higher in the CAD group when compared with the control group. In addition, the RDW was shown to significantly correlate with the RBC count, hemoglobin level and TG level within the laboratory parameters, and significantly correlate with the FRS. Furthermore, the FRS was predicted by hemoglobin levels, TG and RDW in patients with CAD, and the RDW, TG and hemoglobin levels were found to be potentially useful predictors of future cardiovascular risk events in patients with CAD.

The RDW, as measured by a hematology analyzer, is an index of the variation in RBC size. An elevated RDW indicates immature RBC production in the bone marrow (24); thus, an increased RDW is often observed in patients with hematological diseases, including vitamin B12 or folate deficiency or hemolysis. Therefore, subjects were excluded from the current study if they had a history of anemia, had received a previous RBC transfusion or were receiving treatment for anemia, including supplementary iron, folate or an erythropoiesis-stimulating agent. Although diabetes and hypertension are known factors that influence the RDW (25), in the present study, no statistically significant differences were observed among the diabetic and hypertensive patients with regard to the RDW compared with the non-hypertensive and non-diabetic subjects. The elevated RDW observed in the study group was not considered to result from diabetic or hypertensive conditions.

Several possible mechanisms exist for an elevated RDW in patients with acute pancreatitis. For example, an increased RDW in patients with CAD may be associated with inflammation (26). Persistent inflammation is known to be a principal pathophysiological observation and a poor prognostic factor for patients with CAD (27). A possible correlation between the RDW and the severity and characteristics of inflammation may exist; therefore, a theoretical explanation for the elevated RDW observed in the study group may be an aggravated inflammatory status. A statistically significant difference in CRP levels was observed between the patients with CAD and the control subjects, although the CRP level did not exhibit a significant correlation with the RDW. Chronic subclinical inflammation is a well-established entity that precedes *de novo* cardiovascular events (28–30). The condition may adversely affect erythropoiesis via a number of mechanisms, such as the direct myelosuppression of erythroid precursors, reducing renal erythropoietin production and the bioavailability of iron, increasing erythropoietin resistance in erythroid precursor cell lines, and also through the promotion of cell apoptosis (27). Therefore, anisocytosis may result from inflammation via the release of immature RBCs into the peripheral circulation. Increased RDW values have been found to be independently associated with higher levels of CRP, a known surrogate marker of inflammation, as well as a number of additional inflammatory markers, including interleukin-6 and soluble tumor necrosis factor receptors 1 and 2 (31,32). The current findings, however, conflicted with the observations of Zalawadiya *et al* (33), who found that CRP levels increased linearly with increased RDW quartiles. The precise causes responsible for the difference in the reported association are yet to be elucidated; however, ethnic differences in CAD risk prediction and differences in population characteristics or sample size are hypothesized to play a role.

Mechanisms underlying the association between the RDW and the increase in CAD cardiovascular events remain speculative. However, elevated RDWs have been demonstrated to be associated with increased hemodynamic and oxidative stress, which is characteristic of CAD exacerbation (34). Previously, the concept of RDW as a prognostic marker for patients with CAD, and as a predictor for the development of CAD in patients with cardiovascular disease, has been proposed by a number of studies (15,16). However, the mechanisms underlying these novel observations have not been fully elucidated; thus, further study is required. In line with this perspective, the present study hypothesized that higher levels of RDW were associated with the FRS in patients with CAD.

An increased FRS represents the aggravation of risk in patients with CAD (33). The estimation of heart events is important for the management of patients with CAD in clinical practice. Although the present study had a number of limitations, including the unavailability of data regarding actual cardiovascular events during a set follow-up period, the FRS is a well-established risk assessment tool that has a high degree of concordance between the expected and actual event frequencies, which supports the validity of the study observations. Furthermore, the RDW was shown to significantly correlate with the FRS, which is known to correlate strongly with TG levels, TC and smoking. Therefore, the observations of the present study indicate that this simple measurement of RDW may be useful for estimating an elevated FRS in patients with CAD. Future study should focus on specifically investigating the use of RDW to reclassify patients in the intermediate FRS category.

In conclusion, a potential association was identified between the RDW and an increased FRS, which was suggestive of elevated cardiovascular events in patients with CAD. Furthermore, the present study demonstrated that RDW, a simple and inexpensive test, in collaboration with additional biomarkers, may be a useful method for the assessment of cardiovascular event risk changes in patients with CAD.

## Figures and Tables

**Table I:** Baseline characteristics of the patients and control subjects.

Parameters	CAD patients (n=283)	Control subjects (n=109)	P-value
Gender, male/female (n)	194/89	66/43	0.133
Age (years)^a^	67±10	62±10	<0.001
BMI (kg/cm^2^)^a^	25.2±4.1	23.9±3.9	0.420
Current drinking, yes/no (n)	113/170	45/64	0.770
Current smoking, yes/no (n)	138/145	46/63	0.257
Hypertension, yes/no (n)	195/88	43/66	<0.001
Diabetes, yes/no (n)	57/226	12/97	0.038

a Presented as the mean ± standard deviation.

CAD, coronary artery disease; BMI, body mass index.

**Table II:** Hematological and clinical data of the CAD patients and control subjects.

Parameters	CAD group (n=283)	Control group (n=109)	P-value
WBC (10^3^/μl)^a^	6.60±2.57	5.79±1.56	0.002
Neutrophils (%)^a^	63±11	59±9	<0.001
Lymphocytes (%)^a^	25±9	30±9	<0.001
RBC (10^6^/μl)^a^	4.22±0.62	4.37±0.54	0.030
Hemoglobin (g/l)^a^	128±18	135±31	0.012
Hematocrit (%)^a^	0.49±0.04	0.40±0.04	0.609
MCV (fl/cell)^a^	91±5	92±5	0.303
RDW (%)^a^	13.7±1.8	13.1±1.0	0.001
PLT (10^3^/μl)^a^	182±95	178±56	0.678
Glucose (mmol/l)^a^	5.5±1.4	5.3±1.3	0.186
Urea (mmol/l)^a^	5.8±2.2	5.5±1.9	0.210
Creatinine (μmol/l)^a^	83±27	75±27	0.009
Albumin (g/l)^a^	38±7.8	39±7	0.094
AST (U/l)^b^	21 (17–32)	21 (16–25)	0.176
CK (U/l)^b^	79 (55–158)	73 (54–98)	0.019
CK-MB (U/l)^b^	12 (9–18)	11 (9–14)	0.011
LDH (U/l)^b^	167 (140–215)	157 (131–181)	0.007
TC (mmol/l)^a^	4.23±1.09	4.05±0.88	0.813
TG (mmol/l)^a^	1.45±0.86	1.39±0.78	0.894
HDL-C (mmol/l)^a^	1.00±0.31	1.02±0.22	0.943
LDL-C (mmol/l)^a^	2.45±0.91	2.41±0.82	0.682
CRP (mg/l)^b^	1.60 (0.40–7.35)	0.64 (0.30–1.60)	<0.001
NT-proBNP (pg/ml)^b^	613 (149.8–2295)	416 (95.6–1320)	0.079
FRS^a^	9.0±4.9	6.4±3.9	<0.001

a Presented as the mean ± standard deviation;

b presented as the median or interquartile range.

CAD, coronary artery disease; WBC, white blood cell; RBC, red blood cell; MCV, mean corpuscular volume; RDW, red cell distribution width; PLT, platelet; AST, aspartate aminotransferase; CK, creatine kinase; CK-MB, creatine kinase-MB; LDH, lactate dehydrogenase; TC, total cholesterol; TG, triglycerides; HDL-C, high-density lipoprotein cholesterol; LDL-C, low-density lipoprotein cholesterol; CRP, C-reactive protein; NT-proBNP, N-terminal pro-brain natriuretic peptide; FRS, Framingham risk score.

**Table III:** Hematological and clinical data of all the subjects differentiated by diabetic and hypertensive conditions.

Parameters	Diabetic and hypertensive	Diabetic or hypertensive	Not diabetic or hypertensive	P-value
WBC (10^3^/μl)	6.30±2.37	6.51±2.25	6.40±2.41	0.821
Neutrophils (%)	62±10	63±11	63±11	0.918
Lymphocytes (%)	27±9	26±10	27±10	0.474
RBC (10^6^/μl)	4.35±0.63	4.29±0.64	4.18±0.55	0.060
Hemoglobin (g/l)	135±32	129±20	131±25	0.609
RDW (%)	13.4±1.0	13.5±0.9	13.6±1.2	0.763
Creatinine (μmol/l)	83.8±26.3	83.4±31.3	78.1±26.2	0.245
CK (U/l)	79 (51–137)	77 (54–126)	81 (52–132)	0.090
LDH (U/l)	165 (135–208)	158 (131–202)	164 (137–191)	0.125
CRP (mg/l)	1.32 (0.44–5.10)	1.20 (0.37–4.40)	1.26 (0.46–4.80)	0.962
FRS	8.9±4.2	8.2±4.1	7.9±3.1	0.001

WBC, white blood cell; RBC, red blood cell; RDW, red cell distribution width; CK, creatine kinase; LDH, lactate dehydrogenase; CRP, C-reactive protein; FRS, Framingham risk score.

**Table IV:** Correlation coefficients of hematological and clinical variables with RDW in patients with CAD.

	RDW	
	
Variables	*r*	P-value
Age	0.076	0.212
BMI	0.132	0.355
WBC	−0.072	0.236
Neutrophils	−0.051	0.406
Lymphocytes	0.059	0.329
RBC	−0.133	0.029
Hemoglobin	−0.207	0.001
Hemotocrit	−0.023	0.705
MCV	−0.118	0.052
PLT	0.055	0.370
Glucose	0.040	0.509
Urea	0.062	0.311
Creatinine	0.023	0.703
Albumin	−0.010	0.873
AST	−0.019	0.757
CK	−0.026	0.665
CKMB	−0.005	0.934
LDH	0.001	0.991
TC	0.017	0.775
TG	0.226	<0.001
HDL-C	−0.031	0.608
LDL-C	−0.065	0.290
CRP	−0.017	0.814
NT-proBNP	0.019	0.817
FRS	0.206	<0.001

CAD, coronary artery disease; BMI, body mass index; WBC, white blood cell; RBC, red blood cell; MCV, mean corpuscular volume; RDW, red cell distribution width; PLT, platelet; AST, aspartate aminotransferase; CK, creatine kinase; CK-MB, creatine kinase-MB; LDH, lactate dehydrogenase; TC, total cholesterol; TG, triglycerides; HDL-C, high-density lipoprotein cholesterol; LDL-C, low-density lipoprotein cholesterol; CRP, C-reactive protein; NT-proBNP, N-terminal pro-brain natriuretic peptide; FRS, Framingham risk score.

**Table V:** Stepwise multivariate linear regression for FRS in patients with CAD.

Variables	β coefficient	Standard error	Partial *r*^2^	P-value
RDW	0.076	0.025	0.030	0.003
TG	0.473	0.111	0.059	<0.001
Hemoglobin	−0.019	0.006	0.034	0.001

RDW, red cell distribution width; TG, triglyceride; FRS, Framingham risk score; CAD, coronary artery disease.
